# Supplementary figures and images for: A tissue engineering approach for repairing craniofacial volumetric muscle loss in a sheep following a 2, 4, and 6-month recovery
Source: PLoS One. 2020 Sep 21;15(9):e0239152. doi: 10.1371/journal.pone.0239152 (PMC7505427; doi:10.1371/journal.pone.0239152)

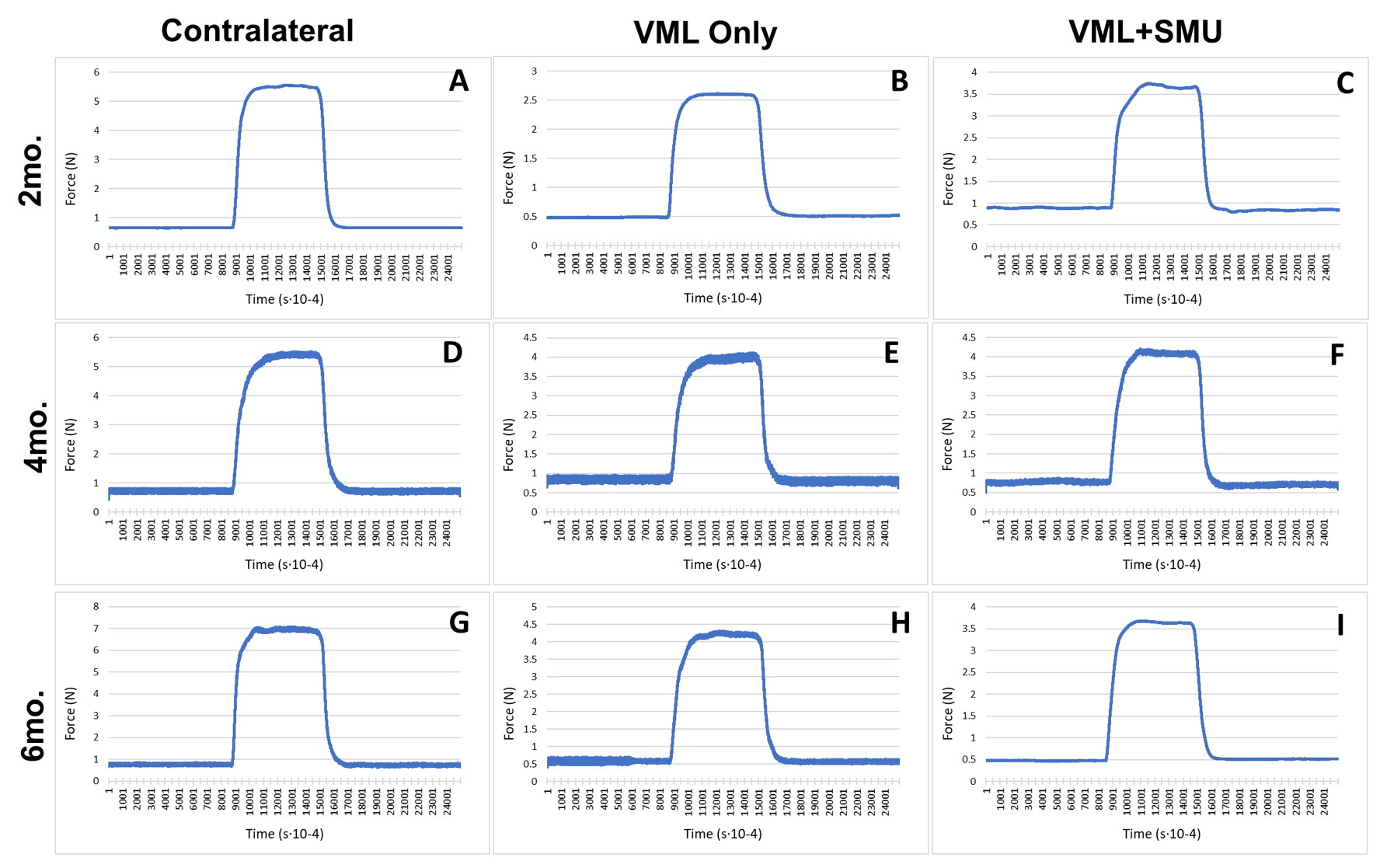

Supplement: S1 Fig — These images depict representative force tracings in response to a tetanic electrical stimulus in the contralateral ZM (A,D,G), the VML Only group (B,E,H), and the VML+SMU group (C,F,I) as well as animals in the 2-month (A-C), 4-month (D-F), and 6-month (G-I) recovery timepoints. (TIF) [file pone.0239152.s001.tif]
