# Supplementary material for: A tissue engineering approach for repairing craniofacial volumetric muscle loss in a sheep following a 2, 4, and 6-month recovery
Source: PLoS One. 2020 Sep 21;15(9):e0239152. doi: 10.1371/journal.pone.0239152 (PMC7505427; doi:10.1371/journal.pone.0239152)
Supplement: Attachment — Submitted filename: PONE-D-20-11394R1 - Response to Reviewers.docx [file pone.0239152.s003.docx]

PONE - D-20-11394R1

Review Comments to the Author

Please use the space provided to explain your answers to the questions above. You may also include additional comments for the author, including concerns about dual publication, research ethics, or publication ethics. (Please upload your review as an attachment if it exceeds 20,000 characters)

**Reviewer #1:** In general, the authors adequately responded to most of my previous queries. Two points remain from my original review that need to be clarified.

**Comment:** Regarding figure 2C, the authors need to clarify how there was a value obtained between the two peak forces that were above the range of testing.

**Response:** To clarify, the equipment responsible for producing the electrical stimulus was what maxed out, not the equipment responsible for measuring the force of the contractions. Our current stimulator can only elicit a maximum current of 1000mA and a frequency of 120Hz. At this current and/or frequency, and due to the size of these large constructs, we were unable to reach maximum tetanic force capabilities of the SMU constructs. Therefore, using this equipment, we maxed out the capabilities of our equipment and were only able to elicit maximum tetanic force measures in two constructs (shown as red points in Figure 2C). So, the maximum forces capabilities of all but two of the SMUs are underestimated in this study. We have added this statement to the methods (Page 9, line 180) and results (Page17, Line 357) section of the paper. We feel that maximum forces that we could elicit with our current equipment is valuable to the reader.

**Comment:** Also, it would be more appropriate to average the SMU forces for those values that are acceptable with the exclusion of the two points.

**Response:** It is actually only on two points that we were able to reach the peak of force production, the rest of the SMUs maxed out the equipment before reaching peak. So, we could just present those two data points as an example of potential maximum tetanic force for the SMUs and eliminate all of the data points that maxed out the equipment.

**Comment:** Regarding the NMJs, it is still unclear why so many of the images for Figure 9 are cross-sections.

**Response:** I am sorry for the confusion. However, the NMJ’s are in Figure 10 and are longitudinal sections, specifically of the repair site. Adjacent to the repair site is native muscle fibers that are indeed oriented longitudinally. However, because the repair site is characterized primarily of disorganized scar tissue, the small muscle fibers present there grew disorganized and are not oriented longitudinally with the rest of the muscle. For this reason, a longitudinal section of the ZM muscle as a whole revealed some fibers and NMJs in the repair site that appeared to be in cross-section. **From the paper:** “Immunostaining of longitudinal sections for acetylcholine receptors (red), synaptic vesicle protein-2 (green), and neurofilament (green) was performed to identify the presence of neuromuscular junctions.”

**Comment:** The claim of disorganized fibers is fine but not well supported by other figures in this manuscript. This does not provide much confidence in this analysis or evaluation, to this reviewer. It would seem the authors are providing the best image they can to make a point and not one that is supportive of the data as a whole.

**Response:** There is a great abundance of scar tissue in the repair site, as noted in figures 8 and 11; however, these are zoomed out images of the entire ZM and from these, the reader is not be able to appreciate the morphology of the repair site. Figures 9 and 10 do show high magnification regions of the repair site and also demonstrate the cross-sectional versus longitudinal phenomenon: even though the images are cross-sections, the disorganized nature of the repair site result in some fibers presenting as longitudinal (see Fig 9D, E,F). The same is true for Fig10 (NMJs) in which a longitudinal section of the muscle as a whole result in some fibers appearing cross-sectional.

**Comment:** The response notes these qualitative analyses was “meant to provide visual support of the denervation hypothesis” however the manuscript states that NMJs were noted as present if there was overlap of pre and post synaptic structures, which would not support analysis of denervation. It only supports the identification of innervated NMJs. Further, how many total NMJs were identified (fully formed or not)?

**Response:** We apologize for the confusion. These were the only NMJs that were able to identified within the repair site. The results specified that “at least one NMJ” was able to be identified. This was supposed to highlight the *lack* of abundance of NMJs in the repair site. The point being made was that there was a stark absence of NMJs in the repair site, suggesting that the repair site is widely denervated. This was clarified in the results, page 31, line 671 and 675: “However, NMJs were widely absent in the majority of the repair site of these experimental groups and the images shown in Fig 10 depict what were usually the only NMJs present. Additionally, although acetyl choline receptors and neurofilament were present at earlier timepoints, small muscle fibers in the injury site do not appear to be reinnervated (no overlapping structures) in the 2mo. and 4mo. recovery groups. Overall, in all experimental groups at all timepoints, there appears to be a lack of innervation in the repair site.”

This was also described in the discussion: “However, even at 6 months, the presence of NMJs in the repair site was very limited, suggesting that a recovery period greater than 6-months is necessary to obtain functional reinnervation of the repair site in this facial VML model.” Again, the point being made was that innervation in the repair site was lacking, both in quantity (only several NMJs identified in the entire recovery site) and quality (the fibers are disorganized and there is no overlap of the pre and post synaptic structures in the 2 or 4 mo. groups).

In my original comment I suggested the discussion on this should be toned down and it was really not. The authors do not provide data that support denervation. Including the qualitative analysis and figures is completely acceptable, but it needs to be discussed as such. Conversely, if the authors are going to keep in the discussion points, they made they should note how many NMJs per muscle were evaluated. If they did evaluate the number of denervated (non-overlapping) and innervated (overlapping) NMJs. Keeping in mind that there are 10,000-20,000 fibers (and subsequent NMJs) per muscle based on the data they provided. Thus, if the authors are only providing evidence of the perhaps 3 NMJs per muscle indicated in figure 9 the comments should be clear.

**Response:** Although there are many muscle fibers in the ZM, we only sought to identify the presence of NMJs in the repair site (with the exception of the contralateral muscle). NMJs overall were widely absent in the repair site. We did not see any NMJs in the 2 or 4mo. groups (specified in the manuscript already) and we only saw several NMJs in the entire repair site of the animals in the 6mo. group. This was specified in the results (see response above for excerpt).

Again, the data provided do not support the discussion of denervation and the authors should rework the discussion of the data directly provided.

**Response**: The qualitative image of the NMJ does not and was not meant to support the argument for denervation. It was solely meant to describe the repair site as lacking innervation at the time of explant. But the fiber grouping results definitely point to denervation and reinnervation. The number of group fibers is quantified (and correlated with percentage of force recovery) in Fig 12. From the figure, you can see that the number of groups fibers present ranged from 8 to 282, and as specified in the manuscript, 70% of the animals had more than 50 grouped fibers.

From the discussion: “The location of our VML injury relative to the MEPs suggests that the muscle resection potentially created a denervation injury in the remaining muscle. This is corroborated by the notable type I fiber grouping, an indication of denervation and subsequent reinnervation [47, 48], that was observed in n=7/10 animals in the 6mo. recovery group.”

Regarding the abundance of type I fibers (Fig 11D), although as a group there is no significant difference from the contralateral, it is apparent that 70% of the animals experienced a large shift in the number of slow fibers, while only 3 animals had a quantity of slow fibers that was within the range of the contralateral. While this were not significant, there is definitely evidence for denervation in these n=7/10 animals, which is also supported by the prevalent type I fiber grouping in these same animals. This line was added to the discussion, line 807, “However, 70% of the animals had a quantity of type I fibers that exceeded the highest number exhibited by the contralateral.”

3) Regarding clustering and denervation link, there are a few statements in the discussion that are not clear. On line 795 (marked version) the authors make clear there is a non-significant change but the authors go on to discuss the implication of this. Additionally, the last sentence of this paragraph (lines 801-804) is unclear. The statement in lines 819 and 850 are not supported by the data and should be edited.

Response: We have gone through the entire manuscript and better defined the fiber grouping results as an indication of acute denervation due to the injury and subsequent reinnervation.

Regarding line 795: Although as a group, this shift is not significant, 70% of the animals exhibited a quantity of type I fibers that exceeded the maximum number exhibited by the contralateral. The lack of significance does not suggest there is not a shift in the number of type I fibers, but rather that the shift was not experienced equally by all animals. Much of our data suggests that the animals experienced a differential severity of the initial VML injury, and this is such evidence. The fiber grouping (more than 50 grouped fibers present in 70% of the animals) provides concrete evidence of denervation and subsequent reinnervation in those animals.

The sentence in lines 801-804 was clarified: “There was also as a significant negative correlation between the number of grouped fibers and the percentage of force recovery in the VML Only group such that a larger number of grouped fibers was significantly correlated with a lower percentage of force recovery. This suggests that the denervation evidenced by the notable fiber grouping likely contributed to the total force deficits experienced in the VML Only groups.”

Response: Line 814 - 818: We reworded this section for clarity.

Response: Line 864: The line was edited to read: “Additionally, the results showed evidence of denervation.” We believe that we have presented sufficient evidence of denervation.

**Reviewer #2: (No Response)**
